# Elovanoids are neural resiliency epigenomic regulators targeting histone modifications, DNA methylation, tau phosphorylation, telomere integrity, senescence programming, and dendrite integrity

**DOI:** 10.21203/rs.3.rs-3185942/v1

**Published:** 2023-07-21

**Authors:** Nicolas Bazan, Surjyadipta Bhattacharjee, Sayantani Kala-Bhattacharjee, Alexander Ledet, Pranab Mukherjee

**Affiliations:** Louisiana State University Health New Orleans; Louisiana State University Health New Orleans; Louisiana State University Health New Orleans; Louisiana State University Health New Orleans; Louisiana State University Health New Orleans

**Keywords:** human neuronal/glia cell in primary cultures, neuroprotectin D1, SASP, ferroptosis, erastin

## Abstract

Cellular identity, developmental reorganization, genomic structure modulation, and susceptibility to diseases are determined by epigenomic regulation by multiple signaling interplay. Here we demonstrate that elovanoids (ELVs), mediators derived from very-long-chain polyunsaturated fatty acids (VLC-PUFAs, n-3, C > 28), and their precursors in neurons in culture overcome the damage triggered by oligomeric amyloid-beta (OAβ), erastin (ferroptosis-dependent cell death), or other insults that target epigenomic signaling. We uncover that ELVs counteract damage targeting histones H3K9 and H3K27 methylation and acetylation; tau hyperphosphorylation (pThr181, pThr217, pThr231, and pSer202/pThr205 (AT8)); senescence gene programming (p16INK4a, p27KIP, p21CIP1, and p53); DNA methylation (DNAm) modifying enzymes: TET (DNA hydroxymethylase), DNA methyltransferase, DNA demethylase, and DNAm (5mC) phenotype. Moreover, ELVs revert OAβ-triggered telomere length (TL) attrition as well as upregulation of telomerase reverse transcriptase (TERT) expression fostering dendrite protection and neuronal survival. Thus, ELVs modulate epigenomic resiliency by pleiotropic interrelated signaling.

## Introduction

Neurons, along with other brain cells, implement thought, memory, and behavior. However, they are often exposed to injury, disease, and various insults, such as uncompensated oxidative stress (UOS). Thus, it can be predicted that neurons utilize adaptive responses to preserve function and homeostasis. Histone modifications, DNA methylation (DNAm), tau phosphorylation, senescence gene programming, and sustainment of telomere length (TL) are involved in healthy aging, unsuccessful aging, and pathologies, including age-related epigenomics associated with neurodegenerative diseases, including Alzheimer’s disease (AD)^1,2^.

DNAm, an epigenomics event in AD onset and progression^3,4^, contributes to modulating synaptic plasticity and homeostasis^4^. There is a void in our understanding of how dysregulated responses can be controlled, including which mediators may be engaged—issues critically important to sustaining cognitive decline associated with age and AD.

In previous studies, we identified elovanoids (ELVs), low-abundance, high-potency pro-homeostatic mediators from the omega-3 fatty acid family^5,6^. ELVs are biosynthesized from precursors made by ELOVL4 (elongation of very long chain fatty acids-4), an enzyme selectively expressed in neurons and enriched in the hippocampus^7^. Mutations in the encoding gene of this enzyme are causative of neurological disorders, including mental retardation^7,8^. The ELOVL4 pathway products, very-long-chain polyunsaturated fatty acids (VLC-PUFAs) 32-carbon-n3 (32:6n-3) and 34-carbon-n3 (34:6n-3) yield biologically active stereospecific di-hydroxylated ELV-N32 and ELV-N34, respectively^5,6^. ELV-mediated signaling elicits neuroprotection when cells are exposed to oligomeric amyloid-beta peptide (OAβ)^9^ and other forms of damage, including *in vivo* experimental ischemic stroke^5^. ELVs also counteract OAβ-mediated cytotoxicity, senescence gene programs, and SASP expression in the retina^9^. Moreover, the abundance of sirtuin 1, recognized as a participant in epitranscriptomics^10,11^, is upregulated by ELVs in UOS conditions in neural cells^5^. These observations indicate the importance of ELVs in preserving neuronal integrity. Critically, it remains unclear whether and how the interplay of ELVs would sustain homeostasis, neuronal survival, and epigenomics.

In this article, we report the discovery that in neurons challenged with stressors, ELVs restore histone modifications, DNAm, tau phosphorylation, telomere integrity, senescence programming, and dendrite integrity. It is of interest that dendrites (*dendron*; tree) are targeted since these branches from the neuronal soma conforming to a *tree-like* structure contain ribosomes, endoplasmic reticulum, Golgi apparatus, and active protein-synthesizing activity. Thus, dendrites modulate protein density in response to neuronal inputs and are active participants in synaptic transmission and memory formation by sustaining and sorting out numerous signals arriving from neurons and transferring them to proper circuits^12,13^.

Our findings reveal a novel layer of regulation and the mediators involved that play a role in the pathogenic mechanisms of neurodegeneration. The stressors used are known to be engaged in acute or chronic cell damage that triggers responses that aim to endure, adapt, or restore homeostasis comprising hallmarks of resilience.

## Results

We used primary human neuronal-glial (HNG) cells, differentiated from neuro-progenitor (NHNP) cells, exposed to oligomeric amyloid-beta (OAβ, 10 μM) or erastin (10 μM) (which interferes with cysteine uptake and depletes Glutathione (GSH) by downregulating system X_c_-, (thereby triggering ferroptosis-mediated cell death) to explore protection by ELVs or their precursors (32:6 or 34:6). In some instances, we have also used UOS ([H_2_O_2_ (1200 μM) plus tumor necrosis factor alpha (TNFα) (10 ng/ml)]), oxygen-glucose deprivation (OGD), or N-methyl-D-aspartate (NMDA) (100 μM). Additionally, we have used primary rat hippocampal neurons in tau hyperphosphorylation studies.

### Free fatty acid precursors of ELVs induced dendrite protection

We have previously shown that ELVs are neuroprotective in primary cerebral cortical and hippocampal neurons in culture exposed to UOS, OGD, or NMDA receptor-induced excitotoxicity^5^. To determine whether the precursors of ELV-N32 and ELV-N34, VLC-PUFAs 32:6 and 34:6, protect neurons, we subjected HNG cells to either OAβ (10 μM) or erastin (10 μM) and treated them with 32:6 or 34:6 (500 nM).

**Figure 1:**
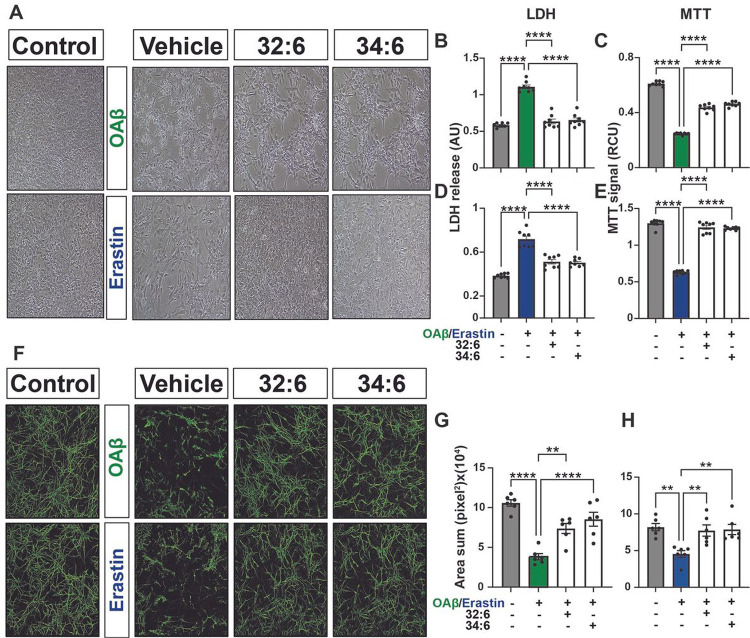
Dendrites protection by precursors of ELVs (32:6 and 34:6). **a,** Representative bright field images (10x mag) of primary human neuronal-glial (HNG) cells in culture challenged with oligomeric amyloid-beta (OAβ) (10 μM) or erastin (10 μM), +/− ELV precursors 32:6 or 34:6 (500 nM). **b-e,** Quantification of neuroprotection by LDH or MTT assay. Data are mean ± SEM of n = 8. *P* values were determined by one-way analysis of variance (ANOVA) at 95% confidence level, followed by Holms Sidak’s post hoc test. ****P ≤ 0.0001. **f,** Mature neurons stained with β-III tubulin showed dendritic damage upon being stressed with either OAβ or erastin +/− 32:6 or 34:6. **g,h,** Quantification of surface area of dendrites with unbiassed image analyses of confocal images of mature neurons stained with β-III tubulin (green). Data are mean ± SEM of n = 6. *P*values determined by one-way analysis of variance (ANOVA) at 95% confidence level, followed by Holms Sidak’s post hoc test. ****P ≤ 0.0001.

Representative brightfield micrographs of HNG cells with different stressors (Fig. 1a) show pronounced dendritic morphology damage with stressors (vehicle) in comparison to untreated (control). 32:6 or 34:6 exerted remarkable neuroprotection and cell survival quantified by lactate dehydrogenase (LDH) and MTT [3-(4,5-dimethylthiazo-2-yl)-2,5-diphenyltetrazolium bromide] assays (Fig. 1b–e). Mature neuron dendrites stained with β-III tubulin (green) displayed severe damage when cells were exposed to OAβ or erastin, and protection took place with either 32:6 or 34:6 (Fig. 1f). Dendrite surface areas, calculated with unbiased image analyses, decrease upon stressing the cells, and protection took place by treatment of the cells with 32:6 or 34:6 (Fig. 1g,h).

Similarly, HNG cells challenged with other stressors—UOS, OGD, or NMDA receptor-induced excitotoxicity —also show pronounced damage caused by the stressors (**Supplementary Fig. 1a**) (vehicle) in comparison to untreated (control), and 32:6 or 34:6 protected dendritic morphology. **Supplementary Fig. 1b-g** depicts the quantification of cell survival by LDH and MTT assays.

**Figure 2:**
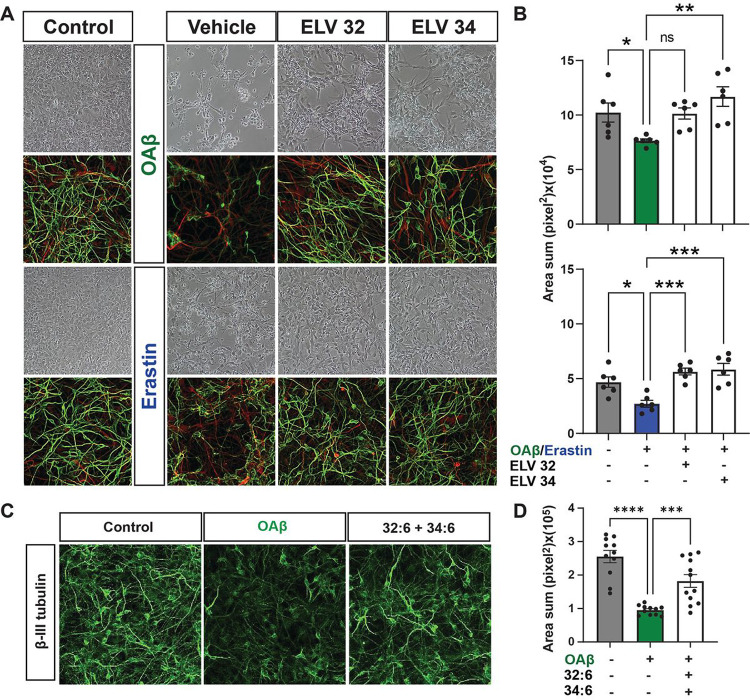
Dendrites protection by ELVs. **a-d and i-l,** Representative bright field images of HNG cells in primary culture. Similarly, **e-h and m-p**show representative immunofluorescent images of HNG cells in primary culture, glial cells stained with glial fibrillary acidic protein (GFAP, red) and mature neuronal dendrites stained with β-III tubulin (green). **a,e,i,m,** Dendrites morphology of unstressed HNG cells (controls); **b,f,** Stressed with OAβ (10 μM) and treated with vehicle (no lipid mediators); **c,g,** Stressed with OAβ (10 μM) plus ELV-N32 methyl ester (Me). **d,h,** Stressed with OAβ (10 μM) plus ELV-N34 Me. Similarly, **j,n** show the morphology of dendrites stressed with erastin (10 μM) and treated with vehicle (no lipid mediators); **k,o,** Stressed with erastin (10 μM) plus ELV-N32 Me; **l,p,** Stressed with OAβ (10 μM) plus ELV-N34 Me. **q,r,** Damage quantification of dendritic surface area induced by either OAβ or erastin (10 μM) and the protection with ELV-N32 and ELV-N34 as determined by unbiassed image analysis with confocal microscopy and Imaris microscopy image analysis software. **s,** Dendritic Protection of HNG stressed with OAβ (10 μM) plus 32:6 and 34:6 (500 nM) each. **t,**Quantification of neuroprotection, as determined by change in dendritic volume between different treatment conditions. Data are mean ± SD. *P* values were determined by one-way ANOVA, followed by Holms Sidak’s post hoc test. ****P≤ 0.0001.

Moreover, we also exposed HNG cells to either OAβ or erastin +/− ELV-N32 methyl ester (Me) or ELV-N34 Me at a concentration of 200 nM (Fig. 2a). ELVs elicit dendrites protection as evidenced by staining with β-III tubulin (green). OAβ or erastin decreases dendritic surface area assessed by unbiased Olympus CellSens microscopy image analysis software (Fig. 2b) and protected by 32:6 and 34:6 together (250 nM).

### ELVs or NPD1 counteract injury-induced tau hyperphosphorylation and neuronal damage

**Figure 3:**
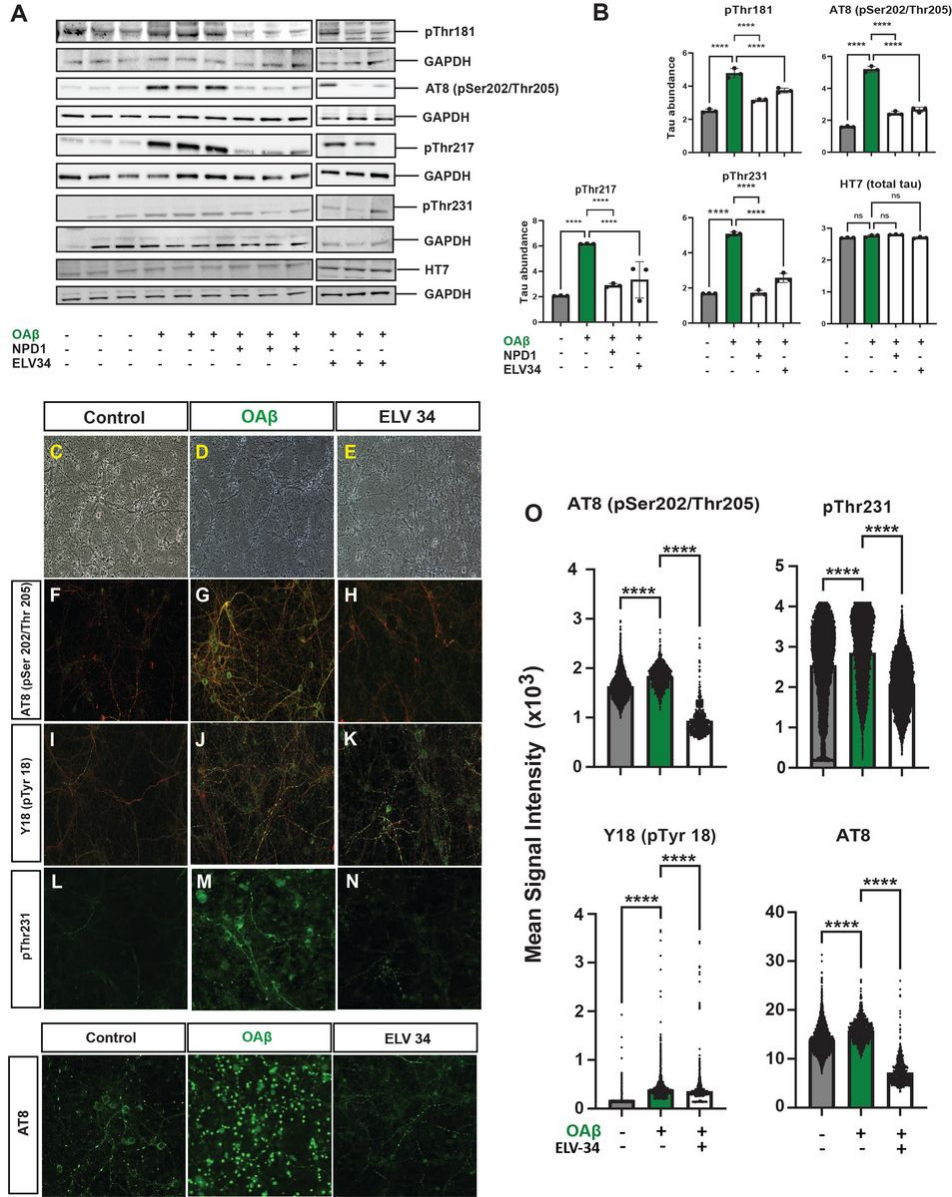
ELVs or NPD1 restore OAβ -induced tau hyperphosphorylation at several phosphorylation sites. **a,** Western blots displaying changes in phosphorylated tau at different phosphorylation residues normalized to GAPDH. There are no changes in total tau (HT7) abundance. **b,** Quantification of phosphorylated tau abundance as determined by densitometry analysis showing downregulation of phosphorylation by NPD1 or ELV-N34 at all phosphorylation sites. **c-e,** Representative bright field images of primary rat hippocampal neurons stressed with OAβ and treated with ELV-N34 (250 nM). **f-n,**Representative confocal micrographs of neuronal cultures stressed with OAβ, treated with ELV-N34 (250 nM) and stained with antibodies for β-III tubulin (red) and phosphorylated tau residues pSer202/pThr205 (AT8), Y18 (pTyr18), or pThr231 (green). **o,** Quantification of change in phosphorylation between unstressed (control), stressed (OAβ), or treated (OAβ stressed + ELV-N34) cells as determined by unbiassed Imaris analysis of green signal intensity. Data are mean ± SEM. *P* values were determined by one-way analysis of variance (ANOVA), followed by Holm Sidak’s post hoc test. ***P ≤ 0.001, ****P≤ 0.0001.

Hyperphosphorylation of neuronal tau, a hallmark of neuronal tauopathies, occurs at key threonine, serine, or tyrosine residues. A key master site is pThr181, which triggers multi-site phosphorylation, thereby fostering assembles of neurofibrillary tangles^14^. Moreover, plasma pThr181 is an early biomarker of MCI and AD^15,16^. Similarly, cerebrospinal fluid pThr217 is a biomarker of AD development^17^. So, we investigated using western blotting key tau phosphorylation residues pThr181, pSer202/pThr205 (AT8), pThr217, pThr231, and total tau (HT7) in primary rat hippocampal neurons stressed with OAβ (10 μM) (Fig. 3a,b). Consistent with previous results, we see hyperphosphorylation of tau at all the residues induced by OAβ and remarkable downregulation of tau phosphorylation by either NPD1 or ELV-N34 Me at 250 nM. There were no changes in total tau HT7 across treatment groups: control, OAβ stressed, NPD1, or ELV. **Supplementary Figs. 2–6** show western blots for all five residues: pThr181, pSer202/pThr205 (AT8), pThr217, pThr231, and total tau (HT7). Also, we used confocal microscopy and unbiassed image analysis to investigate tau phosphorylation at residues pSer202/pThr205 (AT8), Y18 (pTyr18), and pThr231 (Fig. 2c–o). OAβ-induced tau phosphorylation was determined by an increase in the mean signal intensity of those residues, and ELV-N34 Me (250 nM) reduced the mean signal intensity.

### ELVs thwart OAβ or erastin mediated activation of senescence-associated gene transcription: p16^INK4a^, p27^KIP^, p21^CIP1^, andp^53^and of SASP

**Figure 4:**
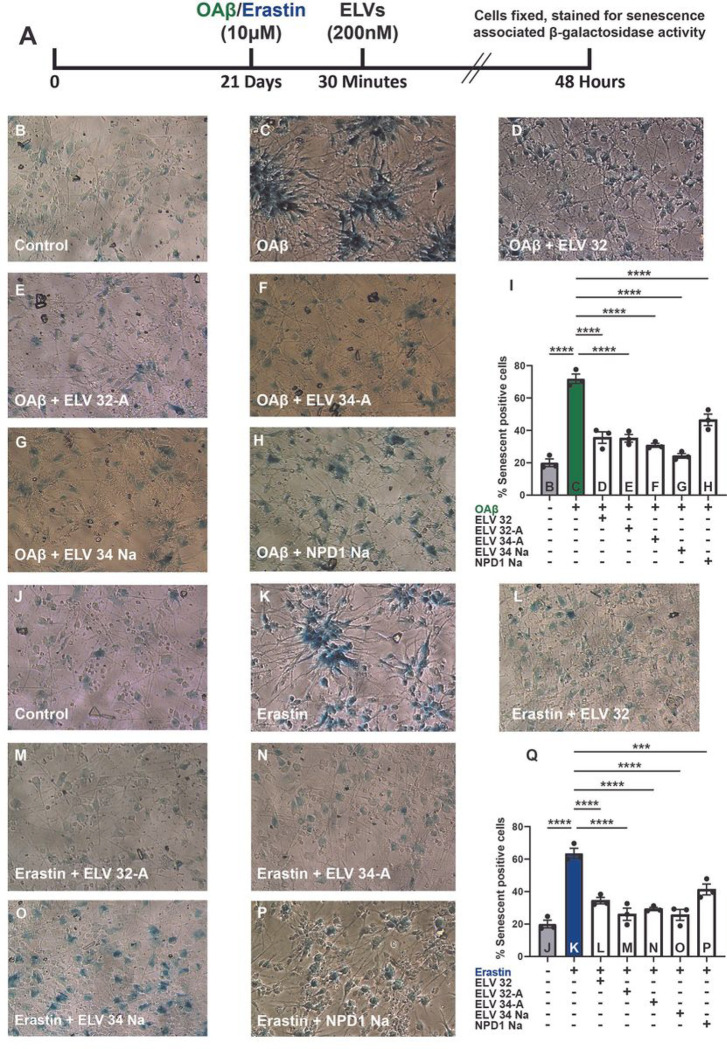
OAβ or erastin-mediated activation of SASP in HNG cells in primary culture counteracted by ELVs. **a,** Experimental design of treatment of HNG co-cultures with lipid mediators. Human neuro-progenitor cells were differentiated into neuronal-glial co-cultures, grown 21 days for maturity, and then stressed with either OAβ or erastin (10 μM). 30 minutes later, lipid mediators were added (200 nM) and incubated for 48 hours, after which cells were fixed and stained for senescence-associated β-galactosidase (SA-β-Gal) activity. **b-h,** Representative images of HNG cells treated with OAβ (10μM) ± ELVs. **j-p,** HNG cells treated with erastin (10μM) ± ELVs. **i,q,** Bar graphs of quantification of the % of β-galactosidase positive cells (senescence-associated secretory phenotype; SASP) and the degree of protection of HNG cells by ELVs treated with either OAβ or erastin, respectively. ELVs decreased positive senescent cells. Data are mean ± SD of n = 3. *P* values were determined by one-way ANOVA, followed by Holm Sidak’s post hoc test. ***P ≤ 0.001, ****P ≤ 0.0001.

Human neuro-progenitor cells differentiated into neuronal-glial co-cultures, grown 21 days for maturity, and then stressed with either OAβ or erastin (10 μM) were used (Fig. 4a). Thirty minutes later, lipid mediators were added (200 nM) and incubated for 48 hours, and then cells were fixed and stained for senescence-associated β-galactosidase (SA-β-Gal). Figure 4b–h displays HNG cells treated with OAβ (10 μM) ± ELVs, and Fig. 4j–p depicts HNG cells treated with erastin (10 μM) ± ELVs. Figure 4i,q illustrates the quantification of β-gal positive cells (senescence-associated secretory phenotype; SASP) and the degree of protection of HNG cells by +/−ELVs treated with either OAβ or erastin, respectively. ELVs or NPD1 decreased positive senescent cells, and we used quantitative PCR (qPCR) to determine the expression of senescence-related genes *p16*^*INK4a*^, *p27*^*KIP*^, *p21*^*CIP1*^, and *p*^*53*^.

**Figure 5:**
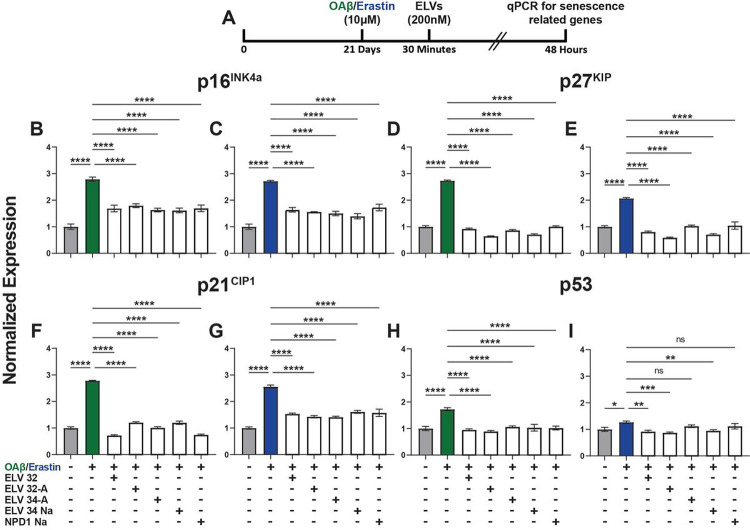
ELVs counteract OAβ or erastin-induced gene transcription of senescence gene programming – *p16*^*INK4a*^, *p27*^*KIP*^, *p21*^*CIP1*^, and *p*^*53*^. **a,** Experimental design of treatment of HNG co-cultures with lipid mediators. Human neuro-progenitor cells were differentiated into neuronal-glial co-cultures, grown 21 days for maturity, and then stressed with either OAβ or erastin (10 μM). 30 minutes later, lipid mediators were added (200 nM) and incubated for 48 hours, after which RNA was extracted, reverse transcribed, and quantitative PCR performed for senescence-related genes. **b-i,** Results for *Cdkn2a* (*p16*^*INK4a*^) (**b,c**), *Cdkn1b* (*p27*^*KIP*^) (**d,e**), *Cdkn1a* (*p21*^*CIP1*^) (**f,g**), and *Trp53* (*p*^53^) (**h,i**). Data are mean ± SD of (n = 3). *P* values were determined by one-way ANOVA, followed by Holm Sidak’s post hoc test. ns = not significant, *P* > 0.05, *P ≤ 0.05, **P ≤ 0.01, ***P ≤ 0.001, ****P ≤ 0.0001.

HNG cells grown until 21 days for maturity were stressed with either OAβ or erastin (10 μM). Thirty minutes later, lipid mediators were added (200 nM) and incubated for 48 hours, after which RNA was extracted and reverse transcribed, and qPCR was performed for senescence-related genes (Fig. 5a). Figure 5 shows results for *Cdkn2a* (*p16*^*INK4a*^) (Fig. 5b,c), *Cdkn1b* (*p27*^*KIP*^) (Fig. 5d,e), *Cdkn1a* (*p21*^*CIP1*^) (Fig. 5f,g), and *Trp53* (*p*^*53*^) (Fig. 5h,i). Both OAβ and erastin induced upregulation of senescence-associated gene transcription, which was counteracted by ELVs or NPD1.

### ELVs reduce OAβ-induced perturbations in DNAm (5-mC), DNA hydroxymethylation (5-hmC) TET activity, DNMT activity, demethylase activity, TL protection, and transcriptional regulation of TERT in HNG cells

**Figure 6:**
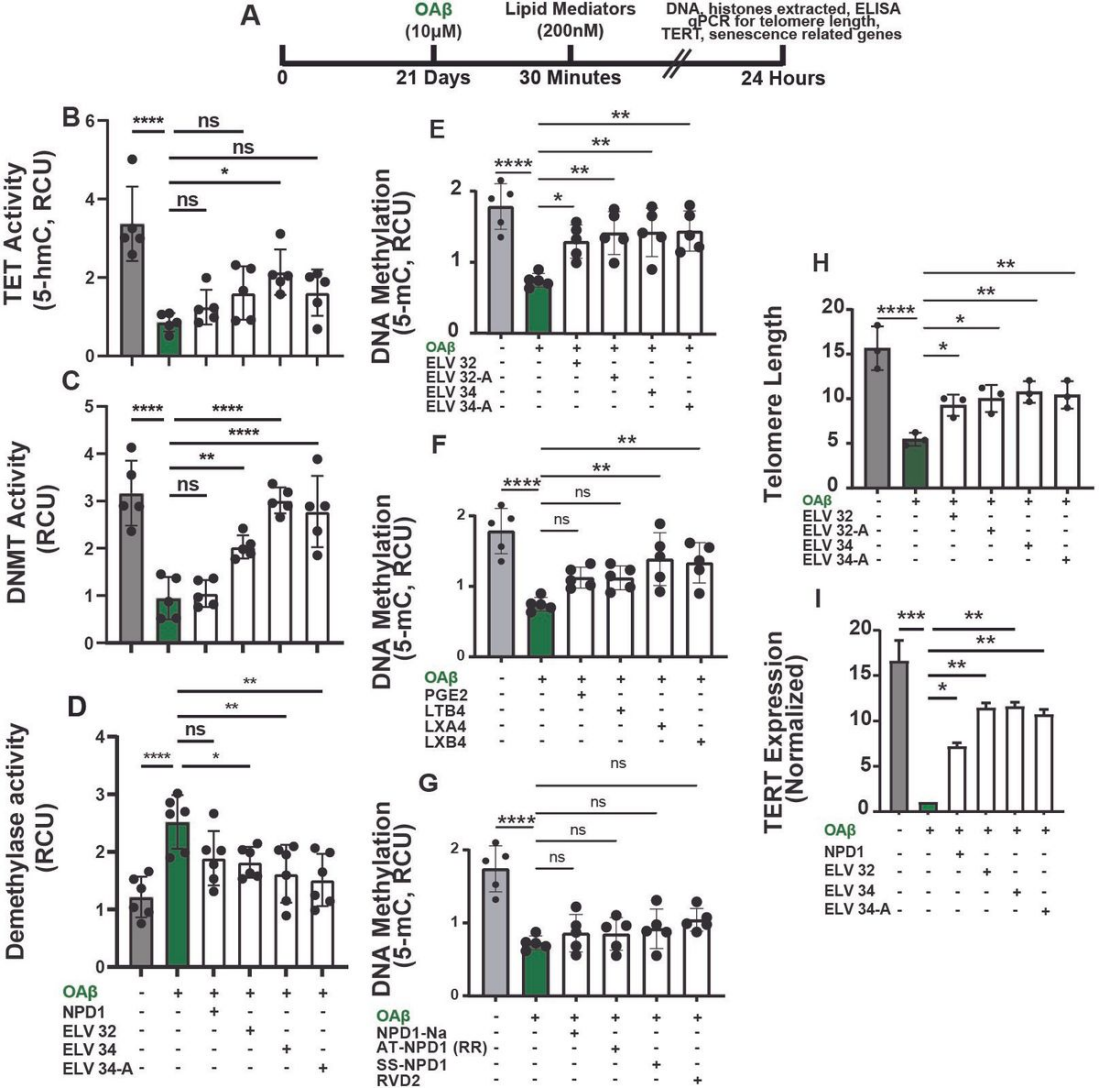
ELVs counteract Oaβ-induced DNA methylation (5-mC), global DNA hydroxymethylation (5-hmC) TET inhibition, DNMT activity, demethylase activity, TL protection, and restoration of TERT expression. We have used primary cultures of HNG cells (**a,i**). **a,**Experimental design of treatment of HNG co-cultures with lipid mediators. Human neuro-progenitor cells were differentiated into neuronal-glial co-cultures, grown 21 days for maturity, and then stressed with OAβ (10 μM) 30 minutes later. Lipid mediators were added (200 nM) and incubated for 24 hours after DNA was extracted for measurement of global DNA methylation (5-mC) and DNA hydroxymethylation (5-hmC), DNMT, or demethylase using ELISA. **b-g,** Global DNA hydroxymethylation, DNMT, demethylase, and DNA methylation ELISA assays were used to determine the relative methylation states of samples treated with vehicle (Control), OAβ (10μM), and various lipid mediators—either NPD1 or ELVs (ELV-N32 Me, ELV-N32 methyl ester acetylenic (Me-A), ELV-N34 Me, ELV-N34 Me-A)—or PGE2, LTB4, LXA4, and LXB4 in (**f**); NPD1-Na, AT-NPD1 (RR), SS-NPD1, and RVD2 (**g**); Control and OAβ values for HNG cells are shared (**e-g**). Data are mean ± SD of n = 5 (**b,c,e-g**), n = 6 (**d**), and n = 3 (**h**). *P* values were determined by one-way ANOVA, followed by Holms Sidak’s post hoc test. ns = not significant, *P*> 0.05, *P ≤ 0.05, **P ≤ 0.01, ***P ≤ 0.001, ****P≤ 0.0001.

Figure 6a shows the experimental layout of the treatment of primary HNG in culture stressed with OAβ. Figure 6e–g illustrates a marked decrease in global DNA methylation (5-mC) levels in HNG cells upon being stressed by OAβ. This DNA hypomethylation is counteracted by ELVs, LXA4, and LXB4. However, NPD1-Na, AT-NPD1, SS-NPD1, or RVD2 could not restore DNAm levels in HNG cells subjected to OAβ (Fig. 6g). As depicted in Fig. 6b, there was inhibition of TET activity in HNG cells when stressed by OAβ resulting in a decrease in DNA hydroxymethylation (5-hmC), which was counteracted by only ELV-N34 Me. Next, we investigated DNA methyltransferase (DNMT) and demethylase activity in HNG cells stressed with OAβ (Fig. 6c,d). There was marked DNA methyltransferase inhibition upon OAβ challenge, which was restored by ELVs. Similarly, the increase in demethylase activity was also counteracted by ELVs, but NPD1 did not alter the TET, DNMT, or demethylase activity. Next, we investigated how the canonical and non-canonical pro-survival functions of telomerase were impacted in HNG cells upon exposure to OAβ. Cells were stressed, lipid mediators were added (200 nM) and incubated for 24 hours, and then DNA was extracted to assess TL per diploid genome copy using qPCR. TL was measured using a quantitative real-time polymerase chain reaction (qPCR) by using an oligomer standard (TTAGGG)_14_. For the samples stressed with OAβ, there is a decrease in the length of telomeres compared to controls, which is restored by treatment with ELVs (200 nM) (Fig. 6h). Telomerase reverse transcriptase (TERT, the catalytic subunit of the telomerase holoenzyme, is the limiting step for telomerase activation and protection of TL. qPCR reveals the downregulation of TERT transcription by OAβ (10μM), which is reversed by ELVs (200 nM) Me and Me-A (Fig. 6i).

### NPD1 or ELVs differentially target H3K27 and H3K9 methylation and acetylation upon challenging HNG cells with OAβ or erastin

**Figure 7:**
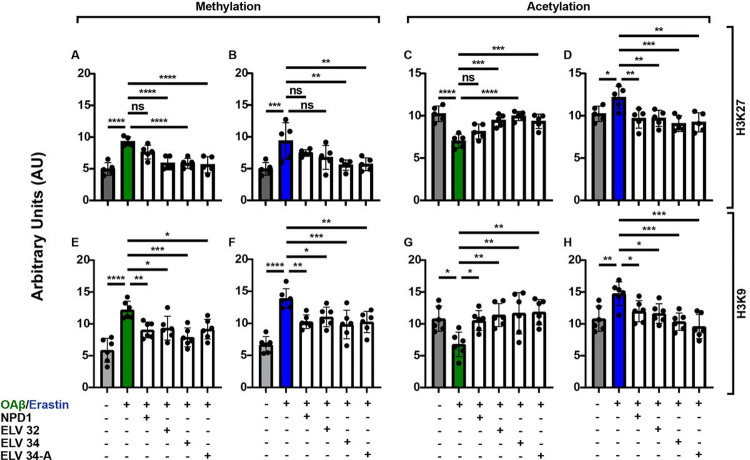
OAβ or erastin-induced H3K27 and H3K9 methylation and acetylation are restored by ELVs. After stressing the HNG cells with OAβ or erastin (10 μM), histones were extracted, and ELISAs were performed to measure H3K27 methylation changes (**a,b**), H3K27 acetylation changes (**c,d**), H3K9 methylation changes (**e,f**), and H3K9 acetylation changes (**g,h**). ELVs counteract H3K27 methylation (**a,b**), but NPD1 had no significant effect on H3K27 methylation. Also, H3K27 acetylation was counteracted by ELVs (**c,d**). Similarly, ELVs counteract H3K9 methylation (**e,f**) and acetylation (**g,h**). Data are mean ± SD of n = 5 (**a,d**) and n = 6 (**e-h**). *P* values were determined by one-way ANOVA, followed by Holms Sidak’s post hoc test. ns = not significant, *P*> 0.05, *P ≤ 0.05, **P ≤ 0.01, ***P ≤ 0.001, ****P≤ 0.0001.

Inflammatory responses and other events associated with longevity involve post-transcriptional histone tail modifications, such as acetylation and methylation of lysine residues, that enable chromatin modifications to activate or repress the transcription of genes. After stressing HNG with OAβ or erastin +/−NPD1 or ELVs, histone modifications were quantified using ELISA assays for methylation and acetylation at H3K9 and H3K27 residues, which specifically are repressor sites for human TERT. Both OAβ or erastin induces hypermethylation of histone 3 at lysine residues 27 and 9 (Fig. 7a,b,e,f), which are counteracted by ELVs or NPD1. However, for H3K27 methylation, NPD1 had no effect. Upon checking for H3K27 and H3K9 acetylation after treatment with OAβ or erastin, we found that OAβ induces hypoacetylation of histone H3 at lysine residues 27 and 9, whereas erastin induces hyperacetylation at H3K27 and H3K9 residues (Fig. 7c,d,g,h). ELVs reversed the changes in hypo- and hyper-acetylation at these residues.

## Discussion

A transcriptomic imbalance during aging and certain pathologies lead to loss of cellular homeostasis and proteostasis, accumulation of protein aggregates, increased genomic instability, mitochondrial dysfunction, telomere attrition, cellular senescence, altered intercellular communication, deregulated nutrient sensing, and a decline in tissue functions^18,19^. These uncontrolled events are linked to epigenomic dysregulation^20,21^ related to histone modifications and DNAm, which regulates gene expression^22,23^ and plays an important role in AD, which presents an accumulation of tau tangles and OAβ plaques^24–29^. Also, several tauopathies and α-synucleinopathies arise, leading to transmission of tau and α-synuclein, which is a mechanism for the progression of neurodegenerative disorders^30^. Interrelated pathways transduce epigenome modulatory signals that include DNAm that modifies cytosine base by DNA methyltransferase enzymes to form 5- methyl-cytosine (5mC). This, in turn, can be modified by the TET proteins (TET1, TET2, TET3) to produce the oxidation products 5-hydroxymethylcytosine (5hmC), 5-formylcytosine (5fC), and 5-carboxylcytosine (5caC). The dysregulation of TET enzymes and 5hmC exhibit differential hydroxymethylation at genes associated with synaptic plasticity, neurogenesis, and neurodevelopment^31–33^, including genes linked to several susceptible AD loci compared to healthy controls^32,34^. Consistent with this, loss-of-function mutations in TET2 have been identified with EOAD patients^33^, and selective genome-wide reduction of 5hmC in neurons leads to hyperphosphorylation of tau and amyloid beta accumulation in the 3xTg-AD mouse model^35^. As demonstrated in Fig. 6, ELVs reduce OAβ-induced perturbations in DNAm (5-mC); in activities of DNA hydroxymethylation (5-hmC) TET, DNMT and demethylase, as well as transcriptional regulation of TERT (catalytic subunit of the telomerase holoenzyme) the limiting step for telomerase activation and protection of telomere length.

In this study, we used different cell injury triggers to explore whether ELVs regulate epigenomic signaling. The stressors used, OAβ 42, a precursor to AD plaques that damages neurons by itself^36^, and erastin to set ferroptosis in motion, limiting the uptake of cystine, consuming GSH, and inhibiting the cystine/glutamate antiporter system (System Xc^−^) and glutathione peroxidase 4 (GPX4)^37^. Ferroptosis is implicated in the amyloidogenic build-up of OAβ in AD due to lipid peroxidation, abnormal iron dynamics, and accumulation in amyloid precursor protein^38–40^. Moreover, UOS, OGD, or NMDA induce cellular excitotoxicity which are also attenuated by ELVs.

In summary, our results show protection of dendritic integrity and cell survival of HNG cells by precursors of ELVs (32:6 and 34:6) or ELV-N32 or ELV-N34 upon being challenged by either OAβ, erastin or other stressors like OGD, NMDA or UOS by precursors 32:6 and 34:6. The results using confocal microscopy, followed by unbiassed image analysis and western blotting demonstrated that ELVs reverse hyperphosphorylation of tau at several key residues important biomarkers of AD—pThr181, pSer202/pThr205 (AT8), pThr217, pThr231—while total tau (HT7) does not change with any of the treatments. Our results on tau hyperphosphorylation—pThr181^41–45^, pThr217^41,46–48^, pThr231^41^, and pSer202/pThr205 (AT8)^41^—are consistent with previous findings that there is hyperphosphorylation of tau at these residues as the cells are challenged by OAβ. Our results show a reversal of hyperphosphorylation by treating the cells with ELVs. This is a novel and critical finding as all these tau residues are correlated with Braak stages II/II and III/IV^41^ and are able to reverse these changes with ELVs. Previous research from our lab has shown that in an Alzheimer’s disease mouse model, *App*^*NL−G−F/NL−G−F*^ (*App* KI), the brain lipidome is preferentially modified during aging when compared to Aβ build-up and pathology^49^. Also, we have shown that intranasal delivery of pro-resolving lipid mediators, rescues memory and improves gamma oscillation impairment in the *App*^*NL−G−F/NL−G−F*^ (*App* KI) model^50^, thereby opening potential avenues of therapeutic intervention and exploration of pro-resolving lipid mediators in AD.

Moreover, we show that ELVs counteract SASP^51,52^ and senescence gene programming^53–55^ induced by OAβ or erastin: p16INK4a^54^, p27KIP, p21CIP1, and p53^55^. ELVs also modulate DNAm modifying enzymes: TET (DNA hydroxymethylase), DNA methyltransferase, DNA demethylase, and DNAm (5mC) phenotype. Histone modifications H3K9 and H3K27 methylation and acetylation play an important role in epigenomic signaling. Hence, we studied these histone modifications in HNG cells stressed with OAβ or erastin and found reversal by ELVs. Upon assessment of TL attrition induced by OAβ, there is protection by ELVs, along with upregulation of TERT expression by treatment with ELVs, which was reduced when cells were stressed with OAβ.

Our study, for the first time, identifies ELVs modulating epigenomic signaling by pleiotropic interrelated mechanisms modulating tau hyperphosphorylation after DNAm, histone modifications, and telomerase activity. We demonstrate that ELVs also counteract senescence gene programming and SASP, suggesting that ELVs can restore epigenomic landscape perturbations that consolidate intrinsic and environmental cues for genome expression and organ functions. With single cell and special transcriptomics/epigenomics^56–61^, we are investigating DNAm^62^ and histone modifications (H3K27me3, H3K27ac or H3K4me3)^63^, to define the interplay of OAβ and tau at synapses^64^ and discern how ELVs may act in resetting epigenomic clock to provide resiliency and conserve the epigenomic signals^65^. Epigenomic perturbations are associated with inflammatory responses, cancer, metabolic disorders (e.g., diabetes, obesity), cardiovascular disorders, aging, and age-related neurodegenerative diseases. The identification of epigenomic regulators would facilitate the understanding and unraveling of functions and dysfunctions of the molecular mechanisms involved. Epigenomic tunings protect or hasten declined cell functions during aging and in age-related disorders. Tau aggregation depends on several post-translational modifications including phosphorylation^41^, participant also of epigenomic modifications. However, the identification of the ELV targeted receptors involved is needed.

Overall, we provide here a characterization of critical targets of ELVs that open insight into regulatory epigenomic landscapes in neurons. We also offer evidence that ELVs target dendrites and neuronal survival. Specifically, we found strong pleiotropic links between histone modifications, DNAm, and other events that are part of epigenomic mechanisms that enable neurons to adjust their expression patterns temporarily or permanently. This study opens opportunities, including developing experimental and clinical interventions that specifically assess the potential for ELVs to enhance resilience for neurodegenerative diseases and/or stroke.

## Online Methods

### Primary cell cultures of neurons

All animals were handled in compliance with National Institutes of Health (NIH) guidelines, and all the experimental protocols were approved by the Institutional Animal Care and Use Committee (IACUC) of LSU Health New Orleans. Primary cultures of rat hippocampal neurons were harvested from 18-day-old embryos (E18) taken from 2-month-old timed pregnant Sprague Dawley (SD) rats (Charles River Laboratories, Worcester, MA, United States) and cultured according to the protocol, as previously described^5^. The cells were plated and maintained in Neurobasal medium (Gibco, Grand Island, NY, United States, Cat#21103049), containing 2% B27 plus supplement (Gibco, Grand Island, NY, United States, Cat#A3582801), along with 0.5 mM Glutamine (Gibco, Grand Island, NY, United States, Cat#25030081) and Penicillin-Streptomycin (50 U/ml) (Gibco, Grand Island, NY, United States, Cat#15070063). The cells were maintained at 37°C, 5% CO_2_ atmosphere in a Heracell Vios 160i incubator (Thermo Scientific, Grand Island, NY, United States). The medium was changed every 3 days, and the neuronal cultures were grown up to 3 weeks until maturity.

Primary cultures of human neuronal-glial (HNG) cells were differentiated from Poietics normal human neuroprogenitor cells (NHNP) obtained from Lonza (Walkersville, MD, United States, Cat#PT-2599). NHNP cells were thawed and maintained as neurospheres in Neural Progenitor Maintenance Medium (NPMM) (Lonza, Walkersville, MD, United States, Cat#CC-3209) until ready to be differentiated. The neurospheres were differentiated and plated in 12-well plates coated with Poly-L-Ornithine Solution (0.01%) (Millipore Sigma, Burlington, MA, Cat#A-004-C) and maintained in Neurobasal medium (Gibco, Grand Island, NY, United States, Cat#21103049), containing 2% B27 plus supplement (Gibco, Grand Island, NY, United States, Cat#A3582801), along with 0.5 mM Glutamine (Gibco, Grand Island, NY, United States, Cat#25030081) and Penicillin-Streptomycin (50 U/ml) (Gibco, Grand Island, NY, United States, Cat#15070063). The cells were maintained at 37°C, 5% CO_2_ atmosphere in a Heracell Vios 160i incubator (Thermo Scientific, Grand Island, NY, United States). The medium was changed every 3 days, and the neuronal cultures were grown up to 3 weeks until maturity, and experiments were done on neurons when they were about 70–75% confluent.

### Lipid mediators

Elovanoids (ELVs) as Na, Methyl ester (Me), and Methyl ester acetylenic (Me-A) and Neuroprotectin D1 (NPD1) were used. In all figure legends, ELVs were represented as ELV-N32 Me (ELV-N32), ELV-N32 Me-A (ELV-N32-A), ELV-N34 Me (ELV-N34), and ELV-N34 Me-A (ELV-N34-A). For DNA methylation (5-mC) and telomere length (TL) measurement, we tested some other lipid mediators such as NPD1-Na, AT-NPD1 (RR), SS-NPD1, RVD2, PGE2, LTB4, LXA4, and LXB4.

### Preparation of OAβ

Aβ (1–42) (HFIP-treated, human, Cat#AS-64129–1) was obtained from AnaSpec, Inc. (Fremont, CA, United States) and resuspended in 1%NH_4_OH/H_2_O (Cat#AS-61322) and DMSO to obtain a concentration of 500 μM and sonicated for 10 min over an ice water bath. Oligomerization of Aβ (1–42) was performed by diluting Aβ (1–42) with sterile PBS in low binding polypropylene microcentrifuge tube for 24 h at 4°C.

### Evaluation of SASP β-galactosidase staining

Cells were stained and visualized for β-galactosidase staining using senescence β-galactosidase staining Kit Cat#9860 from Cell Signaling Technology (Danvers, MA, United States) as per the manufacturer’s protocol. Briefly, HNG cells were washed with PBS and fixed with 4% paraformaldehyde (PFA) solution for 15 min at room temperature. PFA was aspirated thereafter, and cells were washed again with PBS and incubated in 1X staining solution mix (pH 6.0) overnight at 37°C in a dry incubator (no CO_2_). The presence of CO_2_ can cause changes to the pH which may affect staining, resulting in either false positive or negative artifacts. Pictures were taken under brightfield microscope (Nikon Eclipse TS100) 20X magnification after the development of blue color and senescent positive cells counted in 3 different random fields per well.

### DNA and RNA isolation

For *in vitro* experiments, cell culture media was aspirated after treatments, cells were washed with 1X PBS, and cell samples were collected using sterile cell scrapers. DNA and RNA were isolated using the following kits from Qiagen (Hilden, Germany): DNeasy Blood & Tissue Kit (Cat#69504) and RNeasy Plus Mini Kit (Cat#74134), respectively.

### qPCR for telomerase, senescence gene programming, and TL determination

The sequence of the validated primers used for the qPCR experiments is provided in **Supplementary Table 1.** For qPCR reactions, 1 μg of total RNA was used for each sample. 1 μg of total RNA was reverse transcribed using an iScript cDNA Synthesis Kit (Bio-Rad), and the reaction was carried out with BrightGreen 2X qPCR MasterMix (Applied Biological Materials Inc. Richmond, BC, Canada). qPCR was performed in a CFX-384 Real-Time PCR system (Bio-Rad). The expression of target genes was normalized to the geometric mean of housekeeping genes, and relative expression was calculated by the comparative threshold cycle method (ΔΔCT).

### Protein extraction and western blotting analysis

Samples were lysed by RIPA buffer and protein determined by Bradford assay (Bio-Rad,Hercules, CA, USA). After denaturation, 20μl of each medium sample or 30μg of total protein for cell/tissue sample was separated by SDS-PAGE (4–12% gradient) gel (Thermo Fisher Scientific, Waltham, MA, USA) and transferred to nitrocellulose membranes (Bio-Rad). The membranes were blocked by 5% non-fat dry milk in PBST, probed with primary antibodies for 1 h, washed 3x by PBST, probed with secondary antibodies (GE Healthcare, Chicago, IL, USA) for 1 h, and washed 3x by PBST. Protein bands were visualized using an ImageQuant LAS 4000 imaging system (GE Healthcare). Densitometry data were statistically analyzed at 95% confidence level.

### ELISA assays for quantification of 5-mC, 5hmC (TET), DNMT, demethylase, H3K9/H3K27 methylation, and acetylation

For experiments analyzing DNAm (5-mC), TET activity/DNA hydroxymethylation (5-hmC), DNA methyltransferase (DNMT), and demethylase activities along with modifications at lysine 9 and 27 positions of histone H3 (H3K9)/(H3K27) methylation and acetylation, ELISA kits from EpigenTek (Farmingdale, NY, United States) were used according to the manufacturer’s instructions. The following ELISA assays were used in the experiments mentioned in this study: Global DNA methylation (5-mC) ELISA Easy Kit, Cat# P-1030; Epigenase 5mC Hydroxylase TET activity/inhibition Assay Kit, Cat# P-3086; DNA demethylase activity/inhibition Assay Ultra Kit, Cat# P-3008; DNMT activity/inhibition Assay Ultra Kit, Cat# P-3009; EpiQuick Global Pan-Methyl Histone H3-K9 Quantification Kit, Cat# P-3036; EpiQuick Global Tri-Methyl Histone H3-K27 Quantification Kit, Cat# P-3042; EpiQuick Global Acetyl Histone H3-K9 Quantification Kit, Cat# P-4010; and EpiQuick Global Acetyl Histone H3-K27 Quantification Kit, Cat# P-4059. For the extraction of nuclear proteins and histones from neurons and RPE cells, we used these kits: EpiQuick Nuclear Extraction Kit I, Cat# OP-0002; and EpiQuick Total Histone Extraction Kit, Cat# OP-0006.

### Statistics

Data are expressed as mean ± SEM of three or more independent experiments. The data were analyzed by one-way ANOVA followed by Holm-Sidak’s multiple comparisons post hoc test at a 95% confidence level to compare the different groups and considered significant with a *P* < 0.05. Statistical analyses were performed using GraphPad Prism software (9.5.1, San Diego, CA, United States).

## Data Availability

All relevant data are included in the paper. This study did not generate any datasets that were deposited in external repositories. All raw data that support the findings, tools, and reagents will be shared on an unrestricted basis. Concerning this, reasonable requests should be directed to the corresponding author.
